# Supplementary material for: Effect of Fetal Bovine Serum or Basic Fibroblast Growth Factor on Cell Survival and the Proliferation of Neural Stem Cells: The Influence of Homocysteine Treatment
Source: Int J Mol Sci. 2023 Sep 15;24(18):14161. doi: 10.3390/ijms241814161 (PMC10531752; doi:10.3390/ijms241814161)

Full blots with Stain free and β-Actin loading controls

TUBB3 Day 3

TUBB3

Stain Free Blot

Blot Restained for β-actin

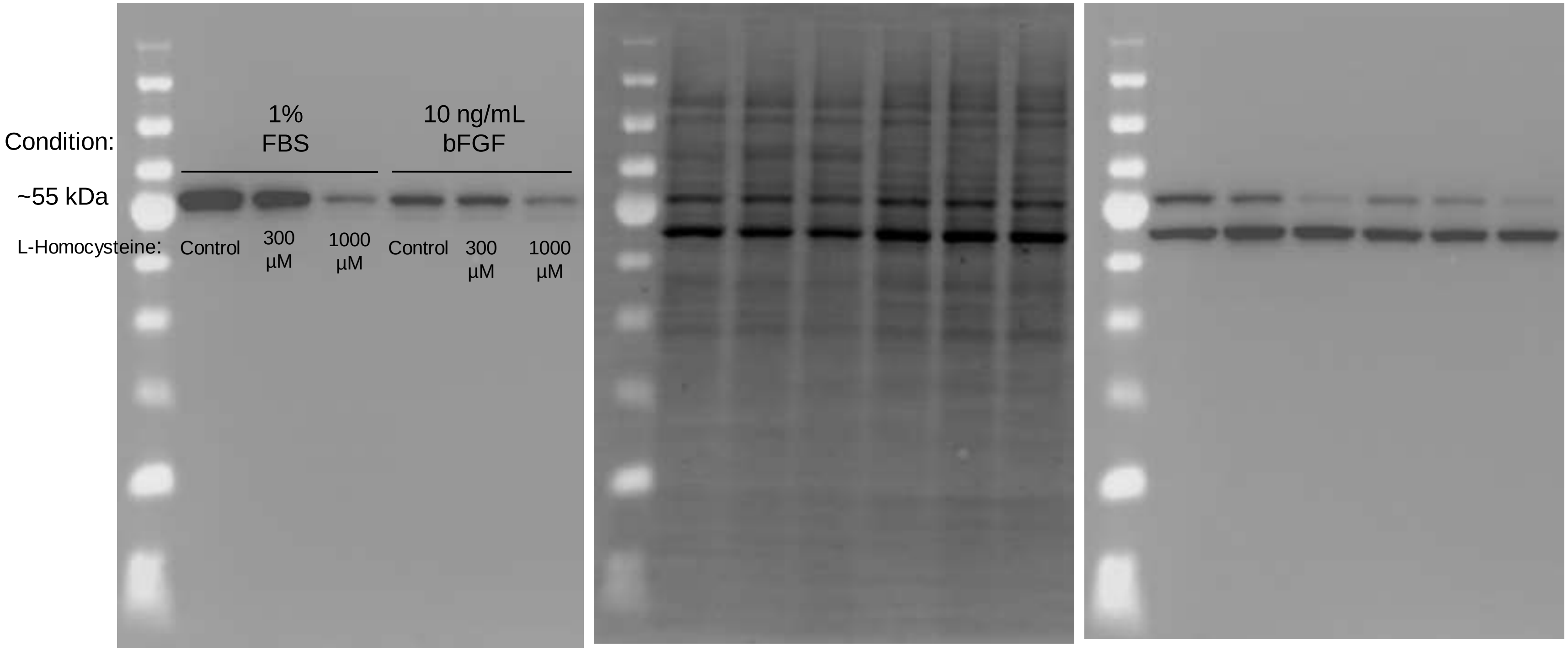

TUBB3 Day 7

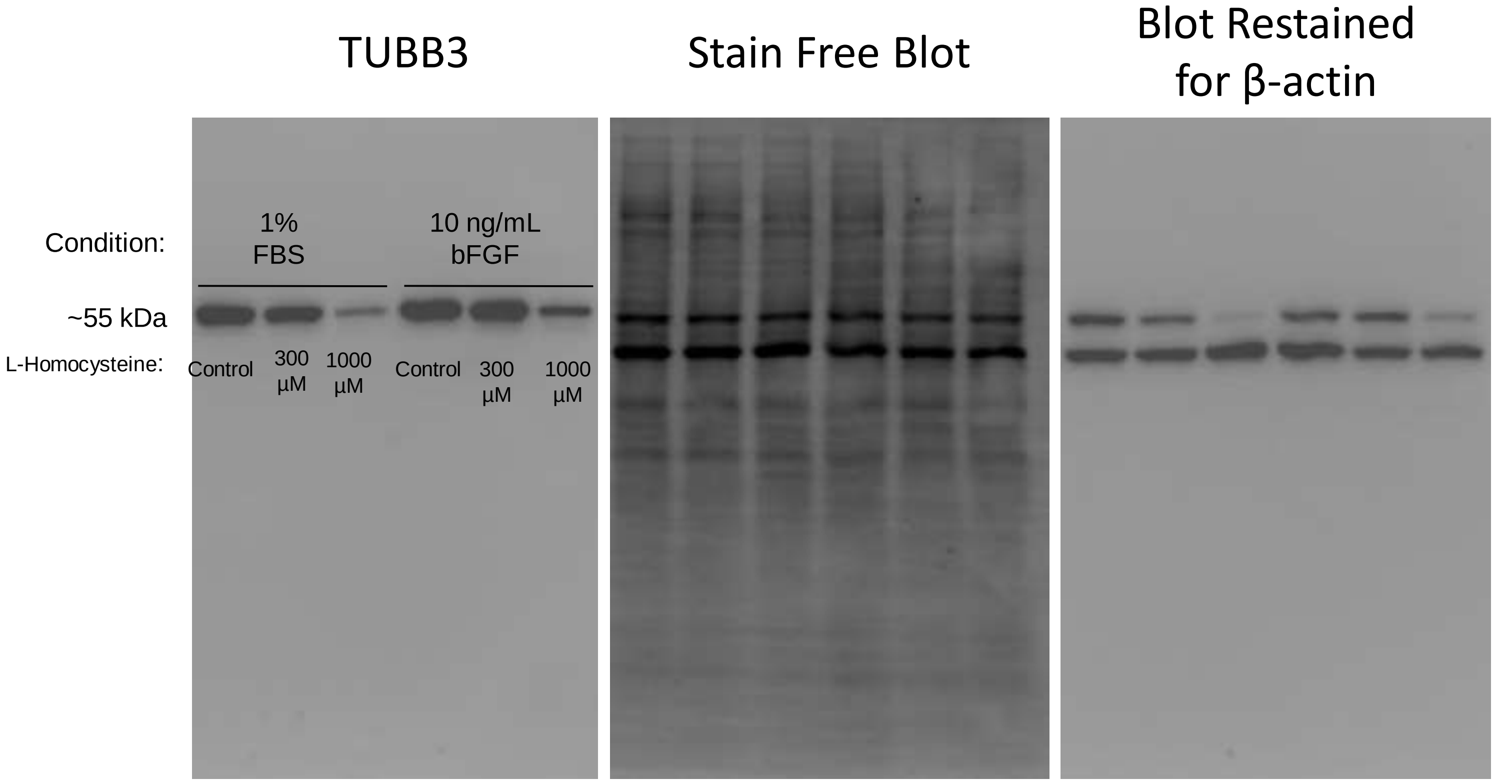
TUBB3
Stain Free Blot
Blot Restained for β-actin
Condition:
1% FBS
10 ng/mL bFGF
~55 kDa
L-Homocysteine:
Control
300 µM
1000 µM
Control
300 µM
1000 µM

GFAP Day 3

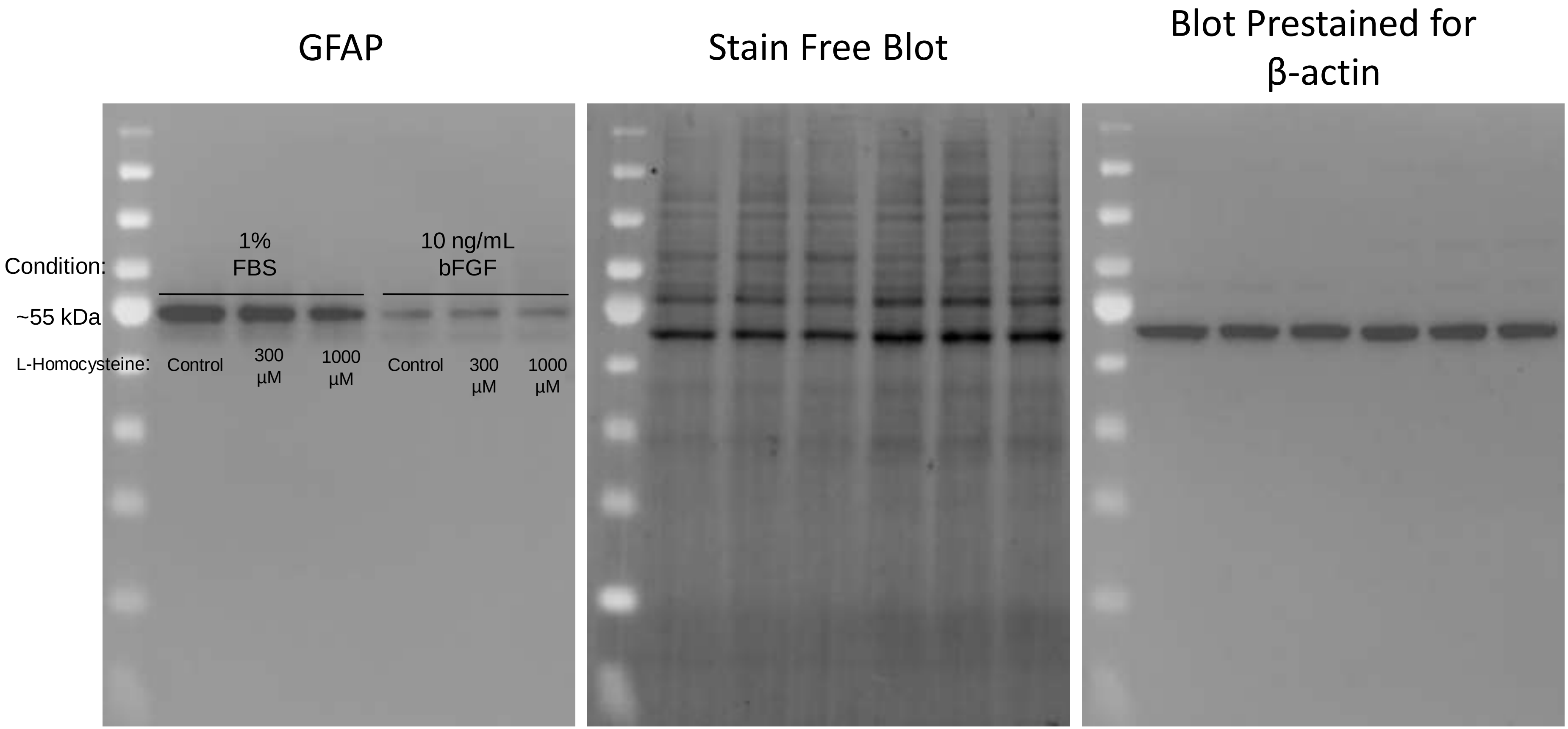
GFAP
Stain Free Blot
Blot Prestained for β-actin
Condition:
1% FBS
10 ng/mL bFGF
~55 kDa
L-Homocysteine:
Control
300 µM
1000 µM
Control
300 µM
1000 µM

GFAP Day 7

GFAP

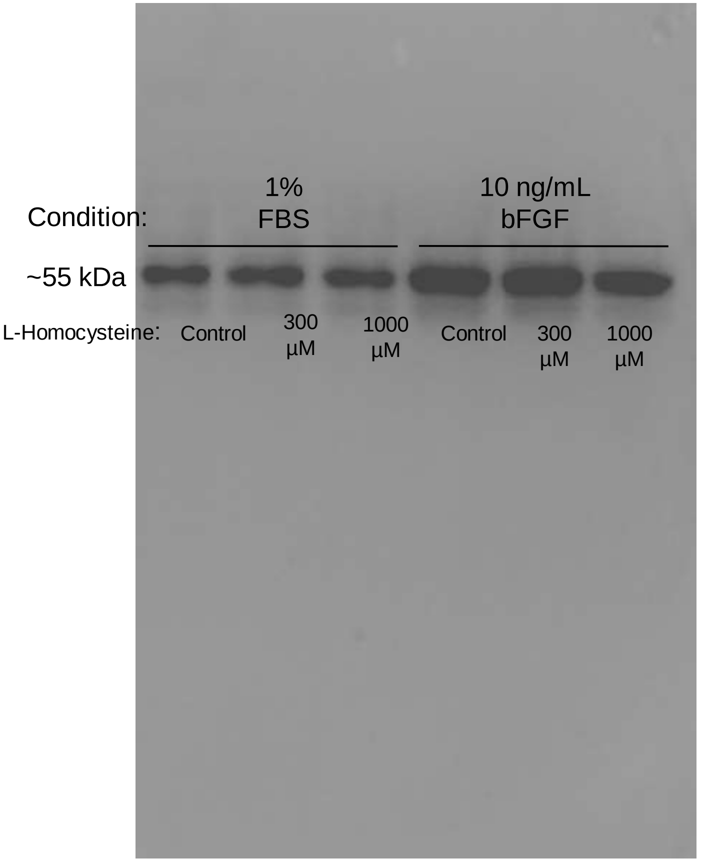

Stain Free Blot

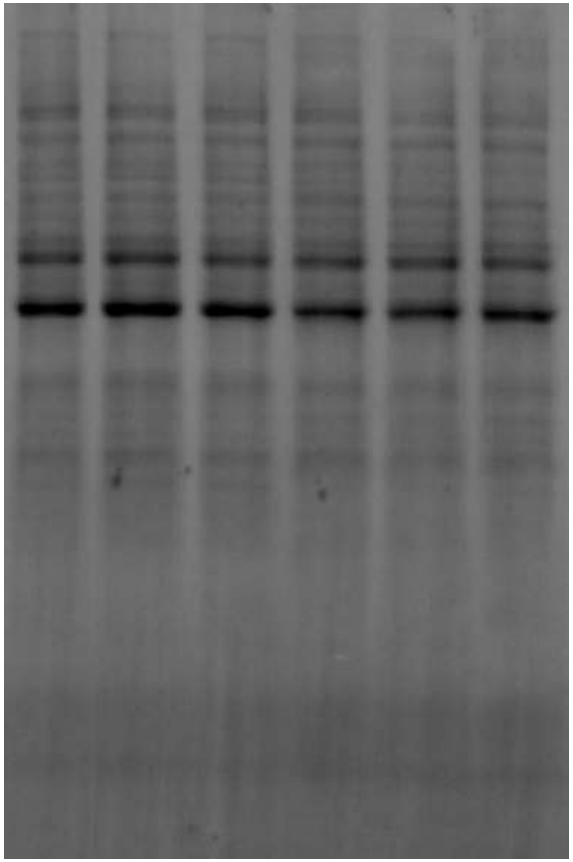

Blot Prestained for β-actin

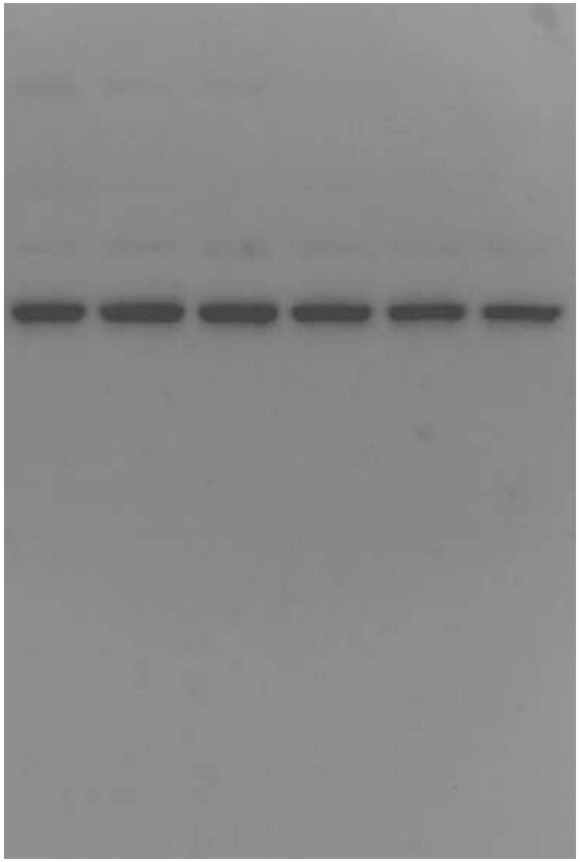

SOX2 Day 3

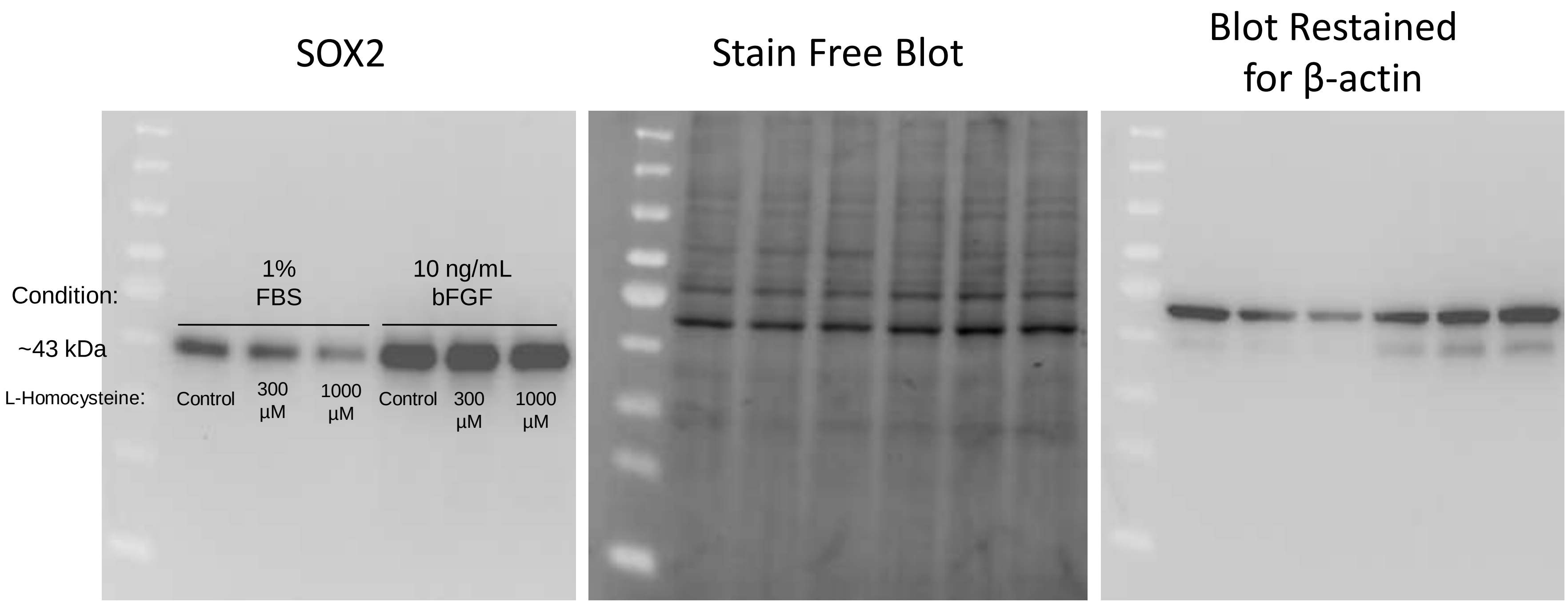
SOX2
Stain Free Blot
Blot Restained for β-actin
Condition:
1% FBS
10 ng/mL bFGF
~43 kDa
L-Homocysteine:
Control
300 µM
1000 µM
Control
300 µM
1000 µM

SOX2 Day 7

SOX2 | Stain Free Blot | Blot Restained for β-actin

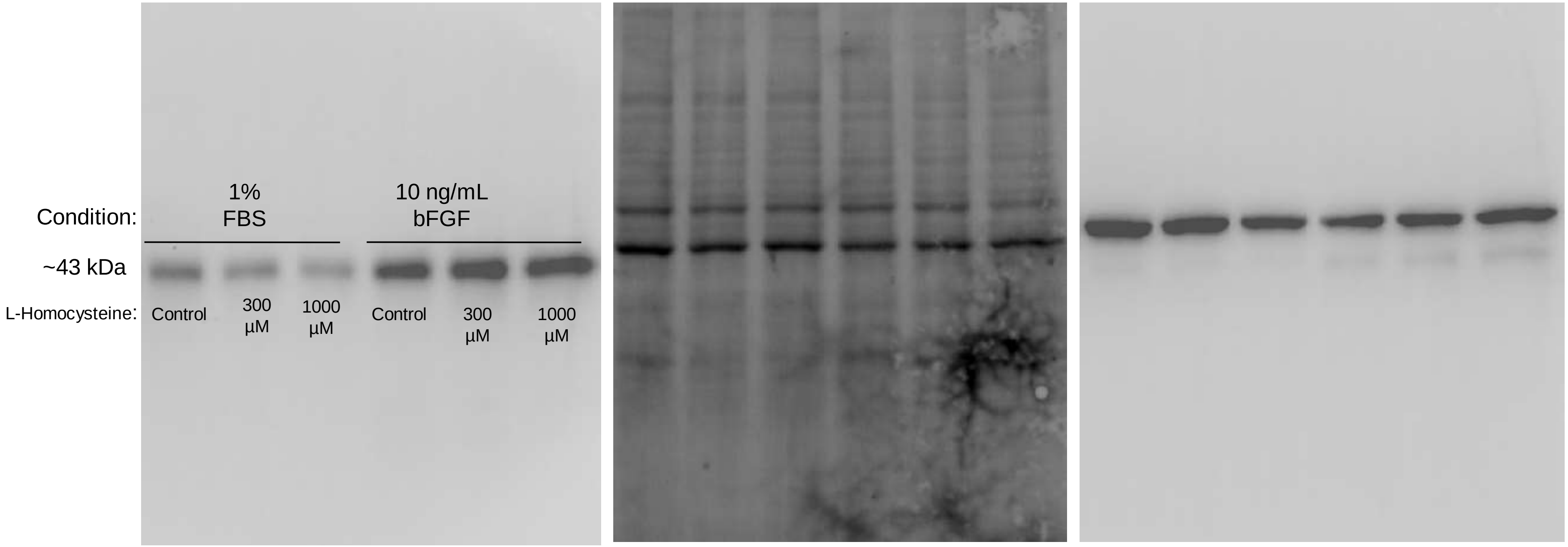

Supplement: Supplementary file 1 [file ijms-24-14161-s001.zip › ijms-2588171-supplementary.pdf]
